# Establishment of the Kuwait Poison Control Center: From concept to operations

**DOI:** 10.1016/j.toxrep.2026.102241

**Published:** 2026-03-22

**Authors:** Abdullatif Aloumi, Fatema Alkandari, Muneera Al Asfour, Robert J. Hoffman

**Affiliations:** a Kuwait Poison Control Center (KPCC), Ministry of Health, Kuwait; b Emergency Medicine Department, Mubarak Al-Kabeer Hospital, Ministry of Health, Kuwait; c Emergency Department, Al-Amiri Hospital, Ministry of Health, Kuwait

**Keywords:** Poisoning, Poison control centers, Emergency medical services, Health care facilities workforce and services, Public health surveillance, Regional medical programs, Kuwait

## Abstract

Poisoning is a significant and largely preventable public health problem, encompassing a wide range of exposures that require specialized toxicological expertise. Poison control centers play a key role in healthcare systems by centralizing clinical toxicological knowledge to support clinical management of poisoned patients, poison surveillance, emergency response, and research efforts. The World Health Organization (WHO) considers the establishment of national poison control centers as a healthcare priority. Concerns over funding, resource, and staff limitations often pose significant barriers to the establishment of poison centers. In Kuwait, the 2012–2016 WHO Country Cooperation Strategy emphasized the urgent need for a national poison control center. However, lack of trained personnel delayed realization of this goal until May 2023. This article describes the establishment of the Kuwait Poison Control Center, detailing early needs assessments, the planning and implementation process, operational structure, and key challenges and achievements encountered during the center’s first two years of operations. It also outlines the center’s current functions and future plans for service expansion and capacity-building.

## Highlights

• Describes phased establishment of Kuwait’s first national poison control center.
• Highlights medical toxicology capacity-building as central to implementation.
• Reports expansion from healthcare-only service to a public 24/7 poison hotline.
• Documents center’s role in mass casualty incidents and national outbreak responses.
• Presents a regional model for poison center development aligned with WHO guidance.

## Graphical Abstract

**Figure:**
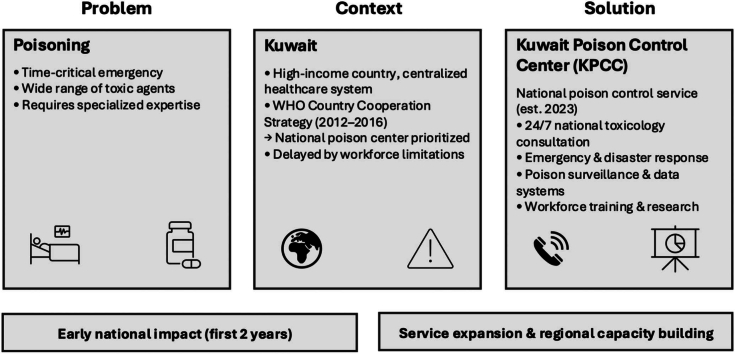

## 1 Introduction

Globally, poisoning is a major public health problem with substantial impacts on morbidity and deaths [1], [2], [3]. In many countries, poisoning is the second leading cause of unintentional injury-related fatality [4]. Importantly, poisoning is both preventable and mitigable. Poison prevention and control efforts, particularly those led by poison control centers, effectively reduce the health burden of poisoning [5], [6], [7], [8]. The specific functions of poison centers can vary considerably between countries or even within the same country; however, recommendations for a standardized core set of poison center activities and capabilities exist [3], [9].

The role of a poison center includes poison management and many other activities [10], [11], [12], [13], [14]; core domains and activities are summarized in Table 1.

**Table 1:** Core activities of a poison control center.

**Domain**	**Activities**
Clinical Toxicology Management	• Provide real-time bedside or telephone consultation services for poisoning emergencies and information requests
Emergency Response and Preparedness	• Respond to all-hazards emergencies (e.g., biological, chemical, radiological or other mass exposure events) • Link with EMS, hospitals, and disaster preparedness agencies
Surveillance and Monitoring	• Monitor the frequency and trends of poisoning cases across the population • Assess outcomes of poisoning cases • Rapid analysis of exposure data (toxicosurveillance) to detect outbreaks • Ensure effective use of such data by public health agencies to assure public safety
Prevention and Policy Development	• Inform and support the development of national poisoning prevention strategies • Provide evidence-based input to policies and regulatory bodies • Translate surveillance findings into preventive actions
Public Education and Access to Information	• Evaluate public education activities related to poisoning • Assess and enhance knowledge of poisoning impact, prevention, and control among members of the public • Establish effective communication with community members regarding poisoning cases • Ensure availability and accessibility of information to the public
Health System Integration and Regulations	• Create provisions for high-quality, culturally competent poison control center services • Establish linkages between PCCs and all parts of the public health system • Ensure consumer protection through continuous monitoring of exposures and regulation of hazardous products • Develop laws, statutes, and regulations that provide for optimal use of PCCs and to protect individuals in the workplace
Workforce Development and Training	• Create and maintain a workforce competent in poison prevention and control • Educate health professionals on subjects related to poisoning
Quality Assurance and Knowledge Generation	• Continuously review and evaluate PCC functions and their efficiency and effectiveness • Identify best practices for PCCs • Contribute to the evidence base for poison prevention and control through the funding and generation of new knowledge

Abbreviations: EMS, emergency medical services; PCC, poison control center.

Specialized clinical toxicology wards first emerged in Europe in the 1940s [15], [16]. In 1953, the first recognized modern poison center was established in Chicago, United States [9]. The World Health Organization (WHO) has recognized the importance of national poison centers since 1994, noting them as a healthcare priority [3]. Nevertheless, many countries struggle to establish and sustain such centers. Recent estimates indicate that only 47% of WHO member states have a national poison center, with gaps noted in African, Eastern Mediterranean, and Western Pacific countries [17]. Common obstacles to establishing and maintaining poison centers include limited resources, particularly funding, and lack of staff trained in toxicology [18], [19]. While Kuwait differs from many lower-resource settings in terms of healthcare infrastructure and funding, these comparisons highlight broader global disparities and the importance of context-specific implementation strategies.

Kuwait is a high-income nation in the Arabian Gulf with a population of 5.02 million [20]. The country maintains a well-developed, state-funded healthcare system overseen by the Ministry of Health (MOH), the primary provider of healthcare services in the country [21]. There are 116 primary care clinics distributed according to population density across all residential areas, with seven tertiary hospitals located within six provinces [22].

Despite the presence of advanced facilities and staff with training and expertise in emergency medicine and critical care, Kuwait had a notable gap in toxicology support and availability of expertise in poison management. The 2012–2016 WHO Country Cooperation Strategy for Kuwait identified that “an important and urgent need is [the] establishment of a poison centre” [23]. Medical toxicology itself is a relatively recent subspecialty, formally recognized by the American Board of Medical Specialties in 1993 [24]. Prior to this, earlier efforts in the United States, including the establishment of the American Academy of Clinical Toxicology (AACT) in 1968 by physicians with interest in and practicing medical toxicology, and certification processes by the American Board of Medical Toxicology between 1975 and 1992, provided an early framework for training and competency standards in medical toxicology [25].

**Fig. 1:**
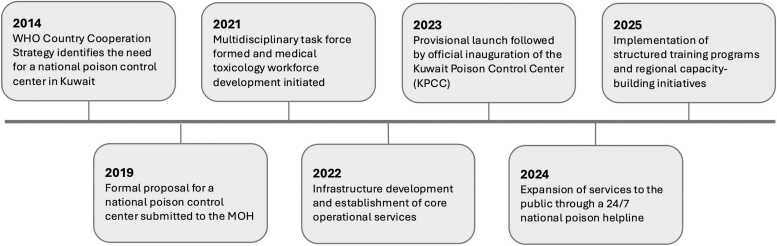
Timeline and key milestones related to the establishment of the Kuwait Poison Control Center (2014–2025). *Abbreviations: SOPs = standard operating procedures; WHO = World Health Organization; MOH = Ministry of Health.*

This article describes the formation of a national poison center in Kuwait, covering the initial needs assessments performed by key bodies in the MOH and Emergency Medicine Council (EMC), the development and execution phases, its operational setup, and the principal challenges faced and milestones achieved during its initial two years. It also highlights ongoing initiatives for service expansion and strategies to extend the center’s impact within Kuwait and the broader Eastern Mediterranean region. The timeline presented in Fig. 1 outlines key milestones in the center’s development and operationalization, from initial concept and needs assessments to infrastructure development, service launch, and ongoing efforts towards service expansion and training development. A phased implementation plan was adopted to ensure quality and service readiness.

## 2 Establishment of the national poison center

### 2.1 Concept

The establishment of the Kuwait Poison Control Center (KPCC) was a multi-phase process spanning several years, marked by strategic planning, coordination, capacity-building, and phased implementation [26]. Initial recognition of the need for a Kuwaiti national poison center was in January 2014 during the WHO Country Cooperation Strategy 2012–2016 mission in Kuwait [23].

### 2.2 Project proposal and task force

Recognizing that medical toxicology requires specialized training, skills, and knowledge that overlaps with but is distinct from emergency medicine, the EMC submitted a proposal for the establishment of a national poison center to the MOH in June 2019. The proposal received strong support. Subsequently, in February 2021, a multidisciplinary task force was formed under the direction of the MOH Minister and Undersecretary. Chaired by the Director of the EMC, this task force was comprised of key national stakeholders, including a medical toxicologist, representatives from various MOH sectors, including pharmacy, laboratory services, information technology, medical stores, drug inspection, legal department, and a representative from the Kuwait University College of Pharmacy.

The task force identified key areas of focus based on the WHO guidelines for the establishment of a poison center and benchmarked models of the American Association of Poison Control Centers [3], [27]. Its primary goals were to:

• Conduct a needs assessment tailored to Kuwait’s population and healthcare system
• Determine the optimal administrative and organizational placement of a poison center within the MOH and the wider healthcare system
• Coordinate with existing MOH departments and external institutions
• Review global poison centers to determine the optimal model for Kuwait
• Determine the ideal location and physical space for the poison center
• Select a telecommunications system capable of supporting 24/7 operations
• Examine administrative, leadership and management aspects related to various poison center functions.

### 2.3 Needs assessments

Comprehensive needs assessments were conducted from August to November 2021. The findings revealed significant gaps in Kuwait’s capacity to manage poisoning cases effectively. Published epidemiological data were scarce, although existing reports indicated a relatively high burden of poisoning, particularly among children [28], [29]. The absence of a formal mechanism for systematically collecting comprehensive epidemiological data on poison exposures and outcomes underscored the need for a centralized surveillance and data collection system [30].

In addition, the assessments also revealed significant variation in the quality of clinical management of poisoning cases, both between hospitals and within specific departments. Availability and appropriate use of antidotes and toxicological laboratory services were inconsistent, leading to disparities in clinical practices. Establishing a poison center staffed by clinicians with expertise in clinical toxicology was deemed a priority to ensure the highest quality of care for poisoned patients.

Key conclusions of needs assessments deemed the following specific deliverables necessary:

• Staffing and training healthcare professionals
• Developing standard operating procedures (SOPs)
• Creating a centralized poison center database and call documentation system
• Supplying and coordinating antidote availability
• Providing specialized toxicology laboratory services

### 2.4 Determination of the optimal poison center model

Needs assessments concluded that a single national poison center was required. Operational definitions of a poison center can vary: In some countries, poison centers function primarily as informational call centers serving healthcare professionals, while others provide inpatient or outpatient clinical care, and still others operate as analytical laboratories without direct clinical services [3], [19], [30], [31], [32], [33]. The task force reviewed global and regional models of poison centers. For Kuwait, the most important function of the national poison center was identified as operating a call center providing expert recommendations to healthcare providers for managing cases of poisoning. Responding to public inquiries was identified as a secondary priority, as access to the healthcare facilities in Kuwait is available across all residential areas.

Based on these primary needs, a call center staffed by physicians, pharmacists, and nurses with training and expertise in medical and clinical toxicology was chosen as the optimal model. Call center operations were established as being necessary 24 h a day, 365 days a year. The center was designed to meet international accreditation standards, such as those of the American Association of Poison Control Centers [27].

### 2.5 Organizational structure and funding

In terms of organizational structure, it was determined that the KPCC would operate as an independent center under the EMC, which itself is a body within the MOH responsible for overseeing the administration and workflow of all emergency departments (EDs) in public hospitals. The KPCC was also designed to serve as a principal component of the MOH’s disaster and mass-gathering preparedness and response efforts, and a key division within MOH interdepartmental committees.

Funding was a consideration in the establishment of a national poison center. Previous research has noted that the primary financial beneficiaries of poison center services are state governments and private insurers, given the substantial cost savings such centers provide [34]. In the case of the KPCC, financing is provided entirely by the MOH [21].

### 2.6 Phased implementation and service provision

#### 2.6.1 Telephone consultations and emergency hotline

Telephone consultations were initiated prior to the establishment of the physical call center. Following completion of his medical toxicology fellowship training in July 2021, the sole clinical toxicologist at the time began receiving consultation requests from various departments, initially limited to critical cases. By February 2022, three clinical toxicologists were hired, and a formal toxicology consultation service was established as a toxicology unit within the Al-Amiri Hospital Emergency Department. This was still restricted to critical cases and those deemed challenging by the treating emergency and critical care physicians. These cases were reviewed daily with the consultant toxicologist, providing case material ideal for teaching medical toxicology, a practice that continues to the present day as part of the KPCC’s daily conferences. In December 2023, prior to the center’s official opening, the KPCC conducted a provisional launch, receiving calls 24/7 from one adult emergency department (ED) and one pediatric ED to ensure readiness of the telecommunication system and to troubleshoot potential issues.

#### 2.6.2 Inpatient clinical services

As complex poisoning cases may require bedside care, the task force first considered establishing a dedicated clinical care unit adjacent to the KPCC. At the initial stage, however, this was deemed too resource-intensive and likely to divert focus from the call center. Instead, KPCC staff were credentialed at and granted access to all MOH healthcare facilities to assess patients in person as needed. While this approach would be impractical in many other settings, it is feasible in Kuwait due to the country’s relatively small size and the KPCC’s centralized location, with even the most distant hospital only a 40-minute drive away.

#### 2.6.3 Laboratory services

Management of poisoned patients may require specialized laboratory testing not routinely available in all medical centers. To address gaps in essential laboratory investigations, the KPCC coordinates with existing MOH laboratories while developing dedicated toxicology testing capabilities.

Toxicology laboratory testing is structured into tiers based on urgency and clinical impact [35], [36]. Tier 1 tests are essential quantitative assays intended for rapid turnaround time to support acute clinical decision-making [36]. Tier 2 tests include broader or more comprehensive investigations requested on a case-by-case basis, typically with longer turnaround times, as outlined in Table 2. For the KPCC’s purposes, qualitative urine toxicology assays were considered a tier 2 test, as their results are less likely to influence acute clinical management despite being commonly included in tier 1 panels in other settings [36].

**Table 2:** Examples of toxicology laboratory investigations according to tier.

**Tier**	**Description**	**Examples**	**Turnaround time**
Tier 1	Essential tests recommended for ready availability in all hospital EDs Findings often have urgent implications for patient management	Quantitative tests • Acetaminophen • Carbamazepine • Co-oximetry* • Digoxin • Ethanol • Ethylene glycol • Iron • Lithium • Methanol • Methotrexate • Phenobarbital • Salicylates • Theophylline • Valproic acid	1–4 h
Tier 2	Additional or specialized investigations that require advanced equipment or processes Findings are typically confirmative or provide additional information but do not tend to guide immediate management	• Heavy metals • Drug of abuse screening • Unknown drug screening with confirmatory tests • Pseudocholinesterase	24 h or longer

Abbreviation: ED = emergency department.

*To determine oxygen saturation, carboxyhemoglobin, and methemoglobin.

The initial review identified deficiencies in assays for ethanol, methanol, and ethylene glycol, making their availability a priority. Dedicated analytical capability for ethanol and toxic alcohol testing was subsequently established.

Since June 2022, following the establishment of the toxicology laboratory at Al-Amiri Hospital, the KPCC has ensured access to tier 1 investigations across all hospitals through a coordinated sample transfer system.

For other tier 2 tests and qualitative urine screening for illicit and unknown drugs, the KPCC coordinates laboratory testing with a pre-existing laboratory at the Kuwait Addiction Treatment Center.

### 2.7 Infrastructure development

#### 2.7.1 Location and physical space

**Fig. 2:**
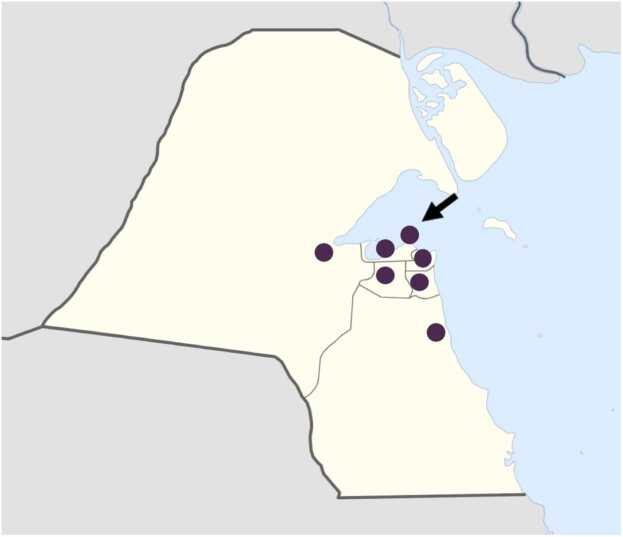
Map of tertiary hospitals in Kuwait indicating the location of Al-Amiri Hospital (arrow).

In May 2022, the task force determined that the poison center should be physically situated within one of the country’s tertiary hospitals to benefit from the logistical support of an established institution. As shown in Fig. 2, Al-Amiri Hospital was selected as the physical base of the KPCC, with development beginning in December 2022.

During the design stage, the call center was the core focus for infrastructure development. The center consists of multiple workstations, each with a computer, telephone and desktop space. Four workstations are currently adequate for handling the typical daily volume of calls; however, the center was designed with 15 additional permanent stations to provide surge capacity. Supporting facilities include a large meeting space for clinical discussions and staff areas.

#### 2.7.2 Telecommunications system

A robust telecommunications system is essential to the fundamental operations of a high functioning call center [3]. Surge capacity was considered when choosing the telephony system. For Kuwait’s population, the switchboard required the capacity to handle 10 simultaneous calls, with up to 50 calls queued. In May 2023, a toll-free hotline number (1804774) was established, with a backup line made available to divert calls. The switchboard is connected to the hospital emergency backup generator in case of electricity failure. All KPCC calls are currently managed by staff fluent in both Arabic and English. Future plans include expanding linguistic capacity to accommodate other spoken languages in Kuwait.

Another effective method for poison center staff to interact with the public and healthcare personnel is via the social messaging platform WhatsApp Messenger® (Meta, Menlo Park, California, United States). WhatsApp® is the most widely used social messaging platform in Kuwait, with near universal adoption [37]. This is particularly useful for sharing clinical context related to poisoning cases, including photos of medications, chemicals, or venomous animals, as well as electrocardiograms (ECGs) and printed investigations such as blood gas results not available in patients’ electronic medical records.

After the initial telephone contact and consultation with healthcare callers, WhatsApp® is used to send written confirmation of the recommended patient management plan. The encryption provided by this platform is considered adequate for KPCC use [38], [39]. In the current context, its security, widespread availability, and familiarity, coupled with established use cases, made it an optimal and practical choice [37], [40], [41].

Communication between the KPCC and the Kuwait emergency medical services (EMS) was also deemed essential. This is currently achieved through multiple methods, including telephone, radio communication, and ambulance telemetry, with the ability to transmit audio and video, as well as live monitoring of vital signs and ECG readings of patients in the prehospital setting. The KPCC also receives automatic notifications from the EMS system for any poisoning-related incidents, including fires.

#### 2.7.3 Centralized database and call documentation system

The task force reviewed various databases and documentation programs used by poison centers worldwide. These systems were found to vary widely, ranging from costly, high-tech solutions to basic standardized datasheets. In August 2022, a decision was made to develop a customized software solution that serves as both a poison center database and call documentation system with key integrations. This platform gives the KPCC the advantage of a dynamic system that can be adjusted as needed, with personalized data displays and statistical outputs.

#### 2.7.4 Integration with allied information systems

The KPCC began linking data from hospitals and primary care clinics to a single site in December 2022. Currently, KPCC staff have real-time access to patient electronic health records, supporting rapid clinical decision-making and tracking trends in poisoning cases.

### 2.8 Workforce development and training of healthcare professionals

#### 2.8.1 Medical toxicologists

**Fig. 3:**
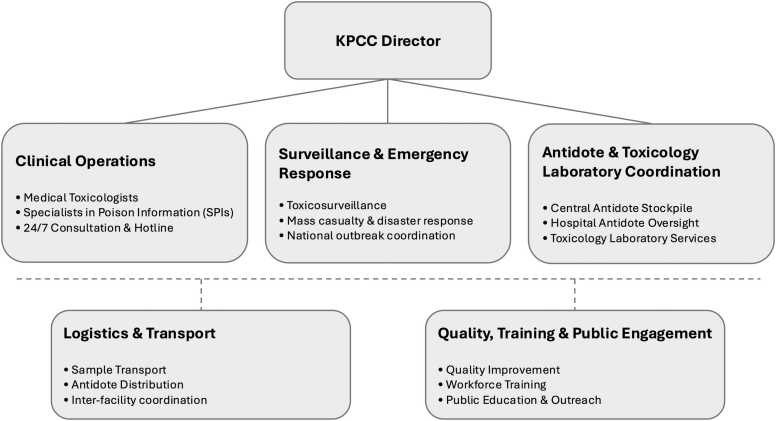
Organizational structure of the Kuwait Poison Control Center. *Abbreviations: KPCC = Kuwait Poison Control Center; SPIs = Specialists in Poison Information.*

One of the greatest challenges encountered during the establishment of the KPCC was staffing with clinicians possessing focused training in clinical toxicology [42]. Prior to the KPCC, there were no formal medical or clinical toxicology training programs in Kuwait. In October 2019, anticipating future needs for a national poison center, the MOH awarded a scholarship to a Kuwaiti emergency medicine physician to pursue fellowship training in medical toxicology abroad. Following completion of his fellowship training in 2021, this toxicologist joined the taskforce and assumed directorship of the KPCC. An external consultant medical toxicologist and three medical toxicologists with doctorate degrees in toxicology were subsequently hired in November 2021 as physician staff with expertise in the specialty. Fig. 3 illustrates the center’s internal organizational structure.

#### 2.8.2 Specialists in poison information

Most of the poison center’s workload consists of handling incoming calls. Effective operation depends on specialized staff trained in poison information [3], [9]. A specialist in poison information (SPI) independently receives calls, documents and collects clinical data, stratifies cases by risk and severity, and provides management recommendations for mild-to-moderate cases according to established guidelines and protocols. For complex or moderate-to-severe cases, the SPI consults with a toxicologist to confirm or initiate management. Most calls are mild-to-moderate and are managed independently by SPIs, who constitute the majority of KPCC staff [9], [43], [44].

Prior to the establishment of the KPCC, the clinical position of SPI did not exist in Kuwait, and the MOH created this class of licensed healthcare provider to facilitate poison center operations. The establishment of a poison center required training of a cadre of specialists to assume this new role. Training SPIs required creating a curriculum and extensive course to provide intensive education. This step, although challenging, was crucial to ensure the sustainability of the KPCC.

Healthcare providers that received KPCC SPI training included junior physicians working in EDs, pharmacists, and nurses. The training was customized to each staff category while still adhering to international standards. It included clinical rotations in adult and pediatric EDs and intensive care units, didactics, and simulated calls. The training was conducted for three months, followed by another three months of probationary period. Since training was based in the KPCC, the SPI training course was conducted on an on-demand basis. In the year prior to the opening of the poison center, 20 staff were trained, 12 of which were initially hired as SPIs. An external expert, a senior SPI from the United States, visited the poison center in November 2022 to evaluate the KPCC training program and assess the SPIs.

#### 2.8.3 External trainees

The KPCC hosts emergency medicine residents during their toxicology clinical rotation and offers an elective toxicology track. Medical students receive an introduction to clinical toxicology in the KPCC during their emergency medicine rotation. Newly hired MOH pharmacists also visit the KPCC during their orientation period. The poison center also contributes to paramedic training, providing instruction on the use of antidotes carried in ambulances.

#### 2.8.4 Future training initiatives

To ensure long-term sustainability, the KPCC has prioritized structured capacity building beyond its initial operational staffing model. In addition to the continued SPI training and professional certification, a two-year Diploma in *Emergency Toxicology Fundamentals* was developed in August 2024, with the first cohort of six residents scheduled to begin in October 2025 under the oversight of the Kuwait Institute for Medical Specializations, the authority responsible for postgraduate medical training in Kuwait. Furthermore, a formal fellowship program in medical toxicology is under development. These initiatives are designed not only to enhance clinical expertise but also to create a sustainable national pipeline of professionals trained in toxicology.

### 2.9 Development of standard operating procedures

To ensure consistent and high-quality clinical care aligned with best practices, the KPCC developed operational policies that included clinical practice guidelines. Common toxicological exposure algorithms were created with input from the external expert to promote consistency in case management, customized to the specific needs of the KPCC. Additional algorithms were designed for special situations, such as mass fire events, carbon monoxide screening, and cyanide poisoning. In April 2024, the MOH tasked the KPCC with creating an SOP for toxicological disasters, including radiation incidents. This document was subsequently adopted across all MOH hospitals. Following this demonstration of subject matter expertise, the KPCC was formally integrated into all MOH disaster plans addressing toxicological emergencies, including fire incidents.

Other targeted SOPs were developed on an as-needed basis. For example, one pediatric ED was recognized to be administrating activated charcoal in cases where it was not indicated. In response, the KPCC delivered an educational presentation and distributed information cards in hospital physician offices and hospital pharmacies outlining the appropriate indications and contraindications for activated charcoal.

#### 2.9.1 Clinical reference materials

The primary reference text used for managing medical toxicology cases is *Goldfrank’s Toxicologic Emergencies*, 11th ed [45]. Additional resources include *UpToDate* (Wolters Kluwer, Alphen aan den Rijn, Netherlands) and Olsen’s *Poisoning and Drug Overdose*, 7th ed., [46]. For day-to-day case management, two toxicology-specific databases are primarily used: Toxbase (UK National Poisons Information Service) and Toxinz (New Zealand National Poisons Center) [47]. Both are English-language, available online, regularly updated, and cover nearly all substance exposures reported to the KPCC.

### 2.10 Antidote supply and coordination

Provision of comprehensive clinical care for poisoned patients requires antidotes. The KPCC determined that ensuring availability of adequate antidote stocks would be one of its core functions. The KPCC maintains an inventory of critical antidotes used in life-threatening situations, commonly used antidotes, and unique, rarely used antidotes that are nonetheless essential [3], [9]. This included: cataloging antidotes; monitoring stocking levels through regular assessments of hospital and health authority inventories; issuing recommendations for administration in actual cases of poisoning, including dosing and frequency instructions; and facilitating antidote sharing across and between facilities.

The first step in developing an antidote supply and coordination system was a national review of all available antidotes [48]. In this process, several essential antidotes absent from the national formulary and stocks were identified, including dimercaprol, edetate calcium disodium, succimer, fomepizole, and hydroxocobalamin. The KPCC requested these be added to the national formulary, and procurement began in May 2022. The review also revealed wide variability in antidote stocking practices across hospitals. To address this, the KPCC developed an antidote-specific formulary and established par levels for common and critical antidotes that should be consistently available across all hospitals.

#### 2.10.1 Central emergency antidote stockpile

To ensure that antidotes would always be accessible when urgently required, a central emergency antidote stockpile was established at the KPCC, available 24 h daily. The default par level for this stockpile is sufficient to treat three adult patients, each with reference weight of 70 kg.

Operationally, if an antidote is urgently required but unavailable at a hospital, the KPCC dispatches the antidote directly to that hospital. If further doses are needed for ongoing treatment, they may be supplied from central MOH stocks. Since the initiation of this system, over 70 patients received life-saving antidotes from the KPCC’s central emergency stockpile.

### 2.11 Public education and poison prevention efforts

An essential role of a poison center is public education and poison prevention [49], [50]. The KPCC conducted over 30 public health outreach events and awareness campaigns in shopping malls, parks, and healthcare facilities.

Other strategies include using social media, television, and radio to spread awareness. The MOH public relations department promoted the KPCC hotline number through its official social media channels. In September 2022, the KPCC launched its own social media presence to engage the public directly.

### 2.12 National and regional coordination

Poison centers rely heavily on collaboration with relevant governmental departments and institutions to ensure successful operations. At the time the KPCC was established, the MOH issued a memorandum to all healthcare facilities, informing them of the services provided by the poison center.

Given its structural integration within the EMC, close coordination with emergency departments (EDs) was essential. Regular feedback from the EDs was therefore critical to improve service delivery. Another key coordination effort involved hyperbaric oxygen therapy (HBOT). In December 2022, Kuwait had a single operational multi-chamber HBOT. The KPCC team visited the site and developed an HBOT protocol for carbon monoxide poisoning, in collaboration with the hospital leadership and relevant departments. This facilitated the transport of 65 patients with severe carbon monoxide poisoning between 2023 and 2024.

The KPCC engaged with multiple external institutions. A formal agreement is in its final stages with the forensics department in the Kuwait Ministry of Interior to streamline access to the forensic laboratory. A chemical disaster drill was conducted with the Weapons of Mass Destruction Defense Command of the Ministry of Defense in December 2024 to establish operational coordination. This educational, hands-on method was found to be the most appropriate when developing initial relationships with external institutions. Similar coordination was implemented with the Kuwait Fire Force and the General Department of Civil Defense. Additional collaborations include research and scholarly projects with the public health sector in the MOH, the Kuwait Oil Company, the Environmental Protection Agency, and Kuwait University.

The KPCC has expanded its regional collaboration efforts with support from the WHO. In February 2024, a meeting of poison center directors from the Eastern Mediterranean Region was convened in Kuwait City to establish a collaborative network. In January 2026, the KPCC hosted the first Eastern Mediterranean Congress, during which the Eastern Mediterranean Poison Control Centers Network was established. The KPCC is also engaged in national disaster preparedness initiatives, including radiation response planning, MOH disaster plan development, and national decontamination preparedness efforts.

## 3 Impacts and lessons

The establishment of a fully operational poison center in Kuwait progressed through three primary phases, each presenting unique challenges and requiring careful planning and dedicated effort to navigate successfully. The preliminary phase involved capacity building by developing a Kuwaiti physician trained in medical toxicology and familiar with poison center operations. This was accomplished by having an emergency physician complete fellowship training abroad. This provided foundational familiarity with poison center operations and specialized expertise in medical toxicology. The second or planning phase included creating a multidisciplinary task force, conducting needs assessments, developing infrastructure, creating SOPs, implementing telecommunications and database systems, and coordinating with hospitals and governmental institutions to ensure integration into the national healthcare system. The final operational stage encompassed staff recruitment and training, launching the 24/7 call center, and initiating clinical, public education, and emergency response services.

### 3.1 Key achievements

The KPCC officially opened in May 2023 under the patronage of the Minister of Health. In its initial phase, services were provided only to healthcare professionals, allowing time to refine protocols and ensure staff readiness for broader operations. By February 2024, the center expanded services to the public through its 24/7 hotline. Key initial benchmarks during the first two years of operations included:

• Management of 2000 poisoning cases in 2023, with a 100% increase in 2024, reaching 4000 cases. This increase is primarily due to greater awareness of the poison center. Call volumes continue to rise, averaging 500 calls per month in 2025.
• Rapid response and management planning for two major mass casualty fire incidents, including one involving 50 patients, six of whom were diverted through the KPCC to receive HBOT for severe carbon monoxide poisoning.
• Launch of structured training and education initiatives for healthcare professionals to develop toxicology knowledge and poisoning management.
• Public awareness campaigns conducted across multiple platforms to enhance community knowledge and safety.
• Participation in over 12 regional and international conferences, resulting in 27 abstracts being published.
• Led the management of the methanol poisoning outbreak in August 2025, which involved early identification and flagging of the outbreak, coordination of care for 162 severely poisoned patients with 50 fatalities, development and implementation of a management plan, organization of fomepizole supply and laboratory investigations, adaptation of treatment protocols to available resources, and contribution to ongoing investigative efforts.

As the KPCC transitions from its initial emergency medicine-led operational phase, we recognize that long-term sustainability requires broader multidisciplinary integration. Collaboration with critical care services (including Extracorporeal Membrane Oxygenation (ECMO) capability), nephrology and extracorporeal therapies, pediatrics, and occupational medicine to ensure comprehensive care for complex toxicological cases is being formalized. In addition, future development will aim to expand training pathways in medical toxicology beyond emergency medicine, including internal medicine, pediatrics, critical care, nephrology, and occupational medicine, reflecting established international models of multidisciplinary toxicology practice.

### 3.2 Challenges and lessons learned

Establishing a poison control center in Kuwait involved challenges, including limited initial public awareness, recruitment of specialized staff, training of staff locally, and adaptation of international models to local needs. Strong leadership, phased implementation, and adherence to WHO standards facilitated operational success [3]. Key lessons learned emphasize the importance of gradual service expansion and continuous staff training.

A national poison center is a vital component of any developed healthcare system, providing evidence-based management plans for poisoned patients, whether at home, in hospital, or elsewhere, 24 h a day, 365 days a year [11], [29]. These centers contribute to preventing hospitalizations, reducing ED and hospital length of stay and associated costs, decreasing morbidity and mortality, and generating data to inform legislation and public campaigns aimed at poison prevention [5], [6], [7], [8]. The expertise of toxicologists and the dynamic function of poison centers make this unit an essential resource for disaster preparedness and emergency response efforts [10], [11], [12], [13], [14], [42]. Daily coordination with different departments and institutions is crucial, whether to supply antidotes, perform laboratory tests, or identify and mitigate emerging poisoning patterns [3], [9].

## 4 Conclusion

The KPCC has addressed an essential gap in Kuwait’s healthcare system by providing timely, expert toxicology guidance that enhances patient care and supports healthcare professionals. Its carefully phased implementation, guided by international best practices, demonstrates a successful model for poison center development in the region. Moving forward, the center aims to expand public education, strengthen toxicovigilance, and strengthen and leverage its data collection activities to inform policy and prevention strategies, further improving national preparedness and patient safety. Furthermore, the KPCC aims to support regional capacity-building by sharing expertise to help other countries establish and strengthen poison control services in line with WHO guidance.

## CRediT authorship contribution statement

**Fatema Alkandari:** Validation, Supervision. **Muneera Al Asfour:** Validation, Supervision. **Hoffman Robert J.:** Writing – review & editing, Supervision, Methodology, Conceptualization. **Abdullatif Aloumi:** Writing – review & editing, Writing – original draft, Validation, Supervision, Project administration, Methodology, Data curation, Conceptualization.

## Funding

This research did not receive any specific grant from funding agencies in the public, commercial, or not-for-profit sectors.

## Declaration of Competing Interest

The authors declare that they have no known competing financial interests or personal relationships that could have appeared to influence the work reported in this paper.

## Data Availability

No data was used for the research described in the article.
